# Patients’ adherence to artemisinin-based combination therapy and healthcare workers’ perception and practice in Savannakhet province, Lao PDR

**DOI:** 10.1186/s41182-018-0125-6

**Published:** 2018-12-22

**Authors:** Emiri Takahashi, Daisuke Nonaka, Moritoshi Iwagami, Vilay Phoutnalong, Ketmany Chanthakoumane, Jun Kobayashi, Tiengkham Pongvongsa, Sengchanh Kounnavong, Bouasy Hongvanthong, Paul T. Brey, Shigeyuki Kano

**Affiliations:** 1 Department of Global Health, School of Health Sciences, Faculty of Medicine, University of the Ryukyus, Okinawa, 903-0215 Japan; 2 SATREPS Project for Parasitic Diseases, Vientiane, Lao People’s Democratic Republic; 3 Department of Tropical Medicine and Malaria, Research Institute, National Center for Global Health and Medicine, Tokyo, 162-8655 Japan; 4 Institut Pasteur du Laos, Ministry of Health, Vientiane, Lao People’s Democratic Republic; 5 Center of Malariology, Parasitology and Entomology, Ministry of Health, Vientiane, Lao People’s Democratic Republic; 6 Lao Tropical and Public Health Institute, Ministry of Health, Vientiane, Lao People’s Democratic Republic; 7 Savannakhet Provincial Health Department, Savannakhet, Lao People’s Democratic Republic; 8 Faculty of Tropical Medicine, Mahidol University, Bangkok, Thailand

## Abstract

**Background:**

Artemisinin resistance in *Plasmodium falciparum* has been spreading across Southeast Asia. Patients’ adherence to artemisinin-based combination therapy (ACT) is critical to avoid expanding this resistance. The objectives of this research were to examine patients’ adherence to ACT for the treatment of uncomplicated malaria and to examine the healthcare workers’ perception of medication adherence and their dispensing practices for malaria patients in Savannakhet province, Lao PDR.

**Methods:**

A prospective observational study of patients and a descriptive study of healthcare workers were conducted in Xepon, Phin, and Nong districts. In the patient study, patients aged 18 years old or older who were prescribed artemether-lumefantrine (AL) at six healthcare facilities between October 2016 and August 2017 were examined. Patient interviews and tablet counts were conducted on the first day of treatment (day 0) and the follow-up day (around day 3). In the healthcare workers study, a self-administered questionnaire survey was conducted.

**Results:**

Of the 54 patients examined, 51 (94.4%) were adherent to the AL regimen. The other three patients stopped medication because they felt better, even though the importance of completing the regimen was explained to all patients when it was prescribed. Among 152 healthcare workers who had ever instructed a malaria patient, 74.3% reported that they occasionally saw a malaria patient who adhered poorly to medication instructions. The healthcare workers perceived the major reasons for poor adherence to be illiteracy and poor understanding of medication instructions by patients. In practice, 27.6% of the healthcare workers did not regularly explain the importance of completing the regimen to patients, and 32.2% did not often or always confirm the patients’ understanding of medication instructions.

**Conclusions:**

Patient adherence to AL was high. The healthcare workers perceived that poor adherence was attributable to the patients, i.e., their poor understanding and illiteracy, which appeared to be related to linguistic differences. However, poor adherence also appeared to be attributable to the healthcare workers, who should tell patients of the importance of completing the AL regimen regardless of their improvement in physical condition and also confirm the patients’ understanding of the instructions.

**Electronic supplementary material:**

The online version of this article (10.1186/s41182-018-0125-6) contains supplementary material, which is available to authorized users.

## Background

Although the morbidity and mortality of malaria have significantly decreased globally [1], resistance to antimalarial drugs has concomitantly been spreading worldwide. Presently, artemisinin-based combination therapies (ACTs), which are the first-line treatment in many countries [2], are the most effective treatment for *Plasmodium falciparum* malaria [3]. The emergence of artemisinin resistance was first confirmed in western Cambodia [4], and it has spread across five countries of Southeast Asia: Cambodia, Lao People’s Democratic Republic (Lao PDR), Myanmar, Thailand, and Vietnam [5, 6]. The spread of artemisinin resistance from Southeast Asia to other regions could trigger a global public health emergency.

Inappropriate use of ACTs can promote artemisinin resistance [7, 8]. Completion of the full regimen is recommended to avoid promoting and spreading this resistance [9, 10]. Pharmacologically, artemisinin clears malaria symptoms quickly so that patients feel better soon after starting the medication [11]. In fact, some patients may stop taking the medication before completing the 3-day regimen [12, 13]. The level of patient adherence to ACT varies greatly depending on the study setting: one systematic review showed the levels of adherence to artemether-lumefantrine (AL) to range from 39 to 96% [14].

Lao PDR is located in Southeast Asia and is bordered by China, Cambodia, Vietnam, Thailand, and Myanmar. As a first-line medication for uncomplicated malaria in Lao PDR, a six-dose, 3-day regimen of AL for adult patients was adopted in 2005. The AL and blood examination are provided free of charge at public healthcare facilities and authorized private pharmacies and by trained village health volunteers [15].

Located in the southern region of Lao PDR, Savannakhet province is one of the most malaria-endemic provinces in the country [16]. According to the Center of Malariology, Parasitology, and Entomology, approximately 90% of all cases in the country in 2016 were reported from the five southernmost provinces including Savannakhet province. Malaria is endemic in 4 of the 15 districts of Savannakhet province: Thapangthong, Phin, Xepon, and Nong.

In recent years, there have been increasing numbers of malaria control and elimination studies targeting these four districts. A targeted malaria elimination study was conducted in Nong district, wherein mass antimalarial administration was integrated with multi-pronged approaches such as the distribution of long-lasting insecticidal nets and strengthening of village health volunteers [17–20]. In addition to these pilot strategies, scientists have shown an increasing interest in the uptake of and adherence to AL. The lack of adherence to the treatment regimen and sub-optimal dosing of AL have been implicated in the increase in artemisinin resistance, which is dependent upon the healthcare workers’ instructions on how to take AL and the patient’s attitude towards completing the prescribed regimen [14, 21].

Nonetheless, few studies have assessed the adherence levels and/or factors associated with the adherence to AL in the region and in Lao PDR specifically. The objectives of this research were to examine patients’ adherence to the AL regimen for the treatment of uncomplicated malaria and to examine the healthcare workers’ perception of medication adherence and their dispensing practices for malaria patients in three of the malaria-endemic districts of Savannakhet province, Lao PDR.

## Methods

**Fig. 1:**
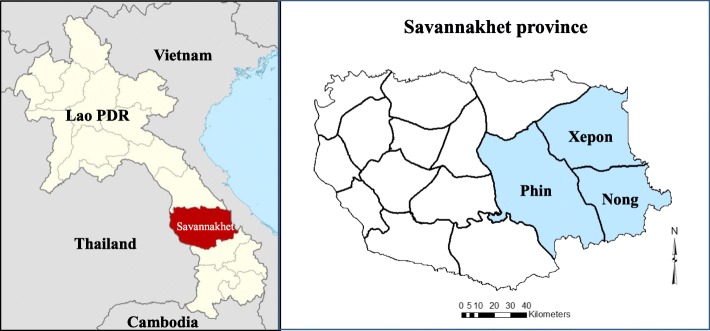
Study districts: Xepon, Phin, and Nong Districts, Savannakhet province, Lao PDR

This research comprised two studies: a prospective observational study of patients and a descriptive study of healthcare workers. Both studies were conducted in Xepon, Phin, and Nong districts of Savannakhet province (Fig. 1).

### Patient study

#### Study site and population

In the patient study, the study healthcare facilities included the district hospital and one health center in each of the three districts, i.e., six healthcare facilities in all. The health centers were selected according to the morbidity of malaria and accessibility of the health center from the district hospital. The study included those patients who visited a healthcare facility during the period between October 2016 and August 2017. Patients who met the following inclusion criteria were included: patients diagnosed as having malaria by a rapid diagnostic test and/or microscopic examination, patients prescribed AL, and patients aged 18 years old or older. Patients with severe or complicated malaria and/or patients who were hospitalized after visiting the outpatient department were excluded. Patients were screened in the outpatient ward on the first day of treatment (day 0) according to the inclusion and exclusion criteria.

In this study, AL was used as the ACT. Depending on the timing of procurement, the use of brand-name drugs (Coartem®) and generic drugs was mixed. The packaging for Coartem® has pictorial directions on medication administration, whereas the generic drug used during the study period has explanations in English on the back side of the information sheet that include the product name, medicinal ingredients, and quantity, which dose is for which day, and sun and moon symbols within a square line frame indicating when each dose (four tablets) should be taken. However, information on which type of drug was used for each patient was not collected.

#### Patient interviews and observations

The surveyors collected the data from each patient via a questionnaire that was originally developed in reference to a previous study [22]. The calculations of minimal sample size were based on the study objective of examining the adherence level. On the basis of the adherence rates to AL that were reported from previous studies (i.e., 64.1%, 65.0%, and 89.5%) [23–25], the adherence rate in the present study was assumed to be 70%. With the 95% confidence interval (CI) for the adherence rate of 70% ± 10%, the required sample size was calculated to be 81 [26]. Considering a drop-out rate of 10%, the sample size was finally determined to be 90. The data collection was planned to continue until the total number of study participants at all study facilities met the sample size. The consecutive sampling method was used.

On the day the medication was first started (day 0), surveyors collected data on the ethnic group of the patient by observing the communication between the healthcare worker and the patient. The surveyors interviewed the patient to collect data on the patient’s contact phone number, occupation, physical condition, symptoms, experience of being prescribed AL, and the content of the medication instructions the patient received. The surveyors also collected data on the patient’s demographics, health insurance, and some clinical information (body temperature, type of malaria, and number of AL tablets prescribed) by referring to the outpatient registry book at the reception desk and the malaria record book in the laboratory.

Surveyors were requested to visit the homes of the patients on day 3 to observe whether any AL tablets were left in the drug packaging sheet and to conduct interviews with the patients. By conducting these interviews, the surveyors collected data on the patients’ practice of taking AL during the treatment course, their knowledge about the AL medication, sociodemographic information, physical condition, and reasons for missing a dose, if any. If the surveyors were unable to visit the home of a patient on day 3, they visited on day 4 as long as the road conditions were good. When the conditions were bad, they were allowed to collect data on the presence or absence of a leftover tablet in the packaging sheet by conducting a telephone interview with the patient.

#### Assessment of adherence

To assess medication adherence, pill count was prioritized when the sheet was available at the home visit. When the surveyor could not visit or the sheet was not available, adherence was assessed based only on the self-report of whether the patient had taken all medicines and had taken them on each day of the regimen.

When the surveyors could visit the patient’s home, in reference to Fogg et al. [27], the patient was classified as “definitely non-adherent” if an AL tablet was left in the packaging sheet, “probably non-adherent” if the sheet was found to be either missing or blank but the patient answered “having not taken all doses,” and “probably adherent” if the sheet was found to be either missing or blank and the patient answered “having taken all of the doses.”. The level of adherence was measured by the adherence rate, which was calculated as the number of patients who were classified as “probably adherent” divided by the total number of patients.

When the surveyors could not visit the patient’s home, the patient was classified as “probably non-adherent” if the patient answered “having not taken all doses” and “probably adherent” if the patient answered “having taken all of the doses” and “taken on each day of the regimen.”

In case the surveyors visited the home of the patient before the regimen was completed, the surveyors observed the discrepancy between the actual number of tablets left and the expected number of the tablets to be left at the time of the observation. If the number was equal to the surveyors’ expectation or the sheet was found to be either missing or blank and the patient answered “having taken the expected doses,” the patient was classified as “probably adherent,” and if the number was more than expected, the patient was classified as “definitely non-adherent.”

#### Healthcare workers observations

The surveyors collected the data on the healthcare worker in charge of the patient via the aforementioned questionnaire. The questionnaire was combined with the “patient interview and observations” form as one questionnaire.

On the day the medication was first started (day 0), the surveyors collected data regarding the healthcare workers in charge of the malaria patient, and at the same time, they collected data on the patient. They observed who gave the medication instructions and how he/she gave the instructions (the means of giving the instructions, the contents of the instructions, and whether the healthcare worker asked if the patient had understood how to take the medicine). If the surveyor arrived at the outpatient department after the patient had already been given the medication instructions, the surveyor interviewed the patient.

The characteristics of the healthcare workers, such as sex, kind of profession, duration of his/her experience in the profession, and ethnic group, were obtained during the interview with the healthcare worker.

### Healthcare workers study

#### Study site and population

In the healthcare workers study, all public healthcare facilities located within the study districts were invited to participate. As a result, all of the invited facilities, namely, 3 district hospitals, 3 district health offices, and 33 health centers, joined the study. Healthcare workers who met the following inclusion criteria were included: healthcare workers who were working for the facilities mentioned above in May 2017 and responded to the questionnaire. Workers such as dentists and hygienists, who were assumed not to give medication instructions to malaria patients, those who had never given medication instructions to outpatients, and those who had not given medication instructions to malaria patients within the past 5 years were excluded.

#### Data collection

A self-administered questionnaire survey was conducted with the healthcare workers between May and August 2017. The questionnaire included questions on sociodemographic information, experience of receiving training on malaria treatment and/or medication adherence, experience of giving a malaria patient medication instructions, experience of seeing malaria outpatients with poor medication adherence, content of the medication instructions they usually provided, and perceptions of responsible persons who managed medication adherence and on possible causes of poor adherence.

#### Statistical analysis

Two groups, healthcare workers who confirmed the patients’ understanding and healthcare workers who did not confirm the patients’ understanding, were compared in terms of sociodemographic characteristics, training experience, content of medication instructions, and perception. The Mann-Whitney *U* test was used for count/continuous variables whereas the chi-square test was used for categorical variables. A *p* value of < 0.05 was considered to indicate statistical significance. All the analyses were performed using SPSS ver. 23.

## Results

### Patient study

#### Patient interviews and observations

During the study period, 269 malaria-positive patients were confirmed. Of them, 91 were prescribed AL and were 18 years old or older. Of these 91 patients examined, 18 patients who met the exclusion criteria were excluded because either the patients were hospitalized and/or they had severe cases of malaria, leaving 73 patients. Of these included patients, 16 were not interviewed on day 0 because of a lack of communication between the surveyors and staff at the study facilities or the surveyors had not been working when the patient consulted. Three other patients were not followed up by day 4: 1 patient was followed up on day 7, 1 patient was not followed up because the patient lived outside of the district and did not have a telephone, and 1 patient was not followed up because of road conditions and not having a telephone (i.e., not followed up on day 2, 3 or 4). As a result, 54 patients completed the interviews on day 0 and on the follow-up day. Of these 54 patients, 14 were interviewed on day 2, 36 on day 3, and 4 on day 4. The patients were planned to be followed up on day 3 or day 4. However, 14 patients were followed up on day 2. Among these 14 patients, 1 patient was classified as “definitely non-adherent” because the patient took only two doses and had left the rest untaken; 1 patient was classified as “probably adherent” even though the patient had one dose (four tablets) left because the surveyor had visited earlier than the scheduled time of the final dose; 7 patients were classified as “probably adherent” because they had taken all prescribed doses as scheduled; and the remaining 5 patients were classified as “probably adherent” although they had taken all of the doses ahead of schedule.

Most of the patients (88.9%) were recruited at the study health centers (Table 1). Over half of the patients (59.3%) were male. The median age was 26 years old (interquartile range [IQR], 19.8 to 35.0 years). Most of the patients (83.3%) belonged to the Mangkong, an ethnic minority group. The most common level of educational attainment was less than primary school (68.5%), followed by primary school (16.7%). Most of the patients (81.5%) were farmers. The type of malaria they contracted included *P. falciparum* in 48 (88.9%), *P. vivax* in 1 (1.9%), and a mix of *P. falciparum* and *P. vivax* in 5 (9.3%). On day 0, 34 patients (63.0%) had a fever equal to or greater than 38 °C. The most common reason for the consultation was headache (100%), followed by nausea (64.8%) and loss of appetite (44.4%). On day 0, 40 (74.1%) felt unwell or moderately unwell, whereas only 3 (5.6%) felt so on the follow-up day (data not shown in table). Almost all of the patients (98.1%) reported that they were explained how to use the medicine by the healthcare worker, and all reported that the healthcare worker confirmed their understanding. Almost all of the patients (94.4%) perceived that the instructions from the healthcare worker were clear, and most (81.5%) had correct knowledge of the dose and timing of the AL.

**Table 1:** Socio-demographic and patient characteristics in the *patient study* (n=54)

Characteristic	n (%)
Study site	
Xepon district hospital	3 (5.6)
Donsavanh health center	22 (40.7)
Phin district hospital	1 (1.9)
Hinsangon health center	0 (0.0)
Nong district hospital	2 (3.7)
Kaison health center	26 (48.1)
Sex	
Male	32 (59.3)
Age (years), median (interquartile range)	26.0 (19.8-35.0)
Ethnic group	
Mangkong	45 (83.3)
Tri	5 (9.3)
Phouthay	2 (3.7)
Lao	2 (3.7)
Educational attainment	
Less than primary school	37 (68.5)
Primary school	9 (16.7)
Secondary school or higher	7 (13.0)
Missing	1 (1.9)
Interpreter on the day of follow-up	
None (patient him/herself)	46 (85.2)
Husband	6 (11.1)
Sister	1 (1.9)
Missing	1 (1.9)
Job	
Farmer	44 (81.5)
Employed (public servant)	2 (3.7)
Others	8 (14.8)
Type of malaria	
*P. falciparum*	48 (88.9)
*P. vivax*	1 (1.9)
Mix of *P. falciparum* and *P. vivax*	5 (9.3)
Fever at the consultation	
≥38°C	34 (63.0)
<38°C	18 (33.3)
Missing	2 (3.7)
Reason for consultation (multiple answers allowed)	
Headache	54 (100)
Nausea	35 (64.8)
Loss of appetite	24 (44.4)
Others	16 (29.6)
Healthcare worker explained to me how to use?	
Yes	53 (98.1)
Missing	1 (1.9)
Healthcare worker confirmed my understanding?	
Yes	54 (100)
Instruction was clear?	
Yes	51 (94.4)
No/Don’t know	2 (3.7)
Missing	1 (1.9)
Answer to the question about correct dose and timing	
Correct	44 (81.5)
Not correct	10 (18.5)
What would you do if you throw up/vomit AL?	
Nothing	2 (3.7)
Take another dose	13 (24.1)
Go back to health facility	34 (63.0)
Do not know	3 (5.6)
Missing	2 (3.7)
This is the first time to take AL?	
Yes	52 (96.3)
No/Do not know	2 (3.7)

On the follow-up day, 3 patients were classified as “definitely non-adherent” whereas the remaining 51 were classified as “probably adherent,” resulting in an adherence rate of 94.4% (51/54, 95% CI 88.3 to 100.6) (Table 2). Among the 3 non-adherent patients, 2 took AL for only 1 day (two doses), and the remaining patient took AL for only 2 days (four doses). They stopped taking the medication because they felt better. They all were Mangkong, had consulted the same health center using health insurance for the poor, and were infected with *P. falciparum* malaria. Although they could correctly answer the question on the doses and the timing of AL, they were unable to appropriately respond to the question on measures against vomiting: To the question “What would you do if you vomit the AL?”, they answered either “Nothing” or “Do not know.” All 3 non-adherent patients reported that they were instructed on how to take AL and the importance of completing the prescribed medicines. They perceived that the instructions were clear.

**Table 2:** Number of adherent and non-adherent patients according to follow-up interview (n = 54)

	Home visit (*n* = 45)	Telephone (*n* = 8)	Face-to-face (*n* = 1)	Total
Non-adherent				
Definitely non-adherent	3	–	0	3
Probably non-adherent	0	0	0	0
Adherent				
Probably adherent	42	8	1	51

#### Healthcare workers observations

Of the 54 healthcare workers who gave medication instructions to the patients, 44 (81.5%) were female (Table 3). Most of the healthcare workers were either Lao (61.1%), which is an ethnic majority group, or Phouthay (25.9%), another ethnic group but speak a language similar to the Lao language. Only 6 (11.1%) of the healthcare workers were Mangkong, an ethnic minority group to which more than 80% of the patients belonged. The most common profession of the person giving medication instructions to the patients was primary health care worker (38.9%), followed by nurse (35.2%), medical doctor (11.1%), and midwife (11.1%). The duration of working experience varied greatly among the healthcare workers: The median duration (IQR) was 10 (2 to 32) years.

**Table 3:** Socio-demographic, characteristics of the healthcare workers in the *patient study* and their medication instructions for the patients (n=54)

Characteristic	n (%)
Sex	
Female	44 (81.5)
Ethnic group	
Lao	33 (61.1)
Phouthay	14 (25.9)
Mangkong	6 (11.1)
Missing	1 (1.9)
Profession	
Primary health care	21 (38.9)
Nurse	19 (35.2)
Medical doctor	6 (11.1)
Midwife	6 (11.1)
Laboratory technician	2 (3.7)
Experience in profession; years, median (interquartile range)	10 (2-32)
Medication instruction (multiple answers allowed)	
Giving oral instruction	54 (100)
Giving written instruction	1 (1.9)
Contents of medication instructions (multiple answers allowed)	
Number of tablets per dose	54 (100)
Number of doses per day	54 (100)
Number of days of the regimen	53 (98.1)
Importance of continuing to take medicines	41 (75.9)
Side effects of medicine	29 (53.7)
Effect of medicine	23 (42.6)
Confirmed understanding of the patient	
Yes	51 (94.4)
No	0 (0.0)
Missing	3 (5.6)

In explaining how to take the medicines, all of the healthcare workers used oral instruction, whereas 1 worker used written instructions together with oral instruction, and all of the healthcare workers explained these requirements to the patients. Almost all of the healthcare workers (98.1%) explained the number of days of the regimen, and most (75.9%) explained the importance of completing the prescribed medicines to the patients. Slightly over half of the healthcare workers (53.7%) explained the side effects of the medicines, and nearly one half (42.6%) explained the effects of the medicine. Almost all of the healthcare workers (94.4%) confirmed the patients’ understanding.

### Healthcare workers study

Of the 267 healthcare workers who were registered at the study healthcare facilities, 248 participated in the study (participation rate 92.9%). Among the 248 responders, those who were assumed not to explain medication use to malaria patients, i.e., five dentists and one hygienist, were excluded. Of the remaining 242 healthcare workers, 84 who had never given medication instructions or had not given medication instructions to malaria patients within the past 5 years and six non-responders to the questionnaire were excluded. Thus, 152 healthcare workers were included in the data analysis (Table 4). Of them, 108 (71.1%) were female and 120 (78.9%) were under the age of 40. The language used at home by most of the participants (96.1%) was Lao. Approximately one half of the participants (51.3%) worked at health centers, and the rest mostly worked at district hospitals (40.1%). The most common profession was nurse (33.6%), followed by medical doctor (27.0%), community midwife (14.5%), and primary health care worker (11.8%). The median (IQR) duration of their working experience was 7 (3 to 12) years. Regarding past experience with training, 101 (66.4%) had received training on malaria, 81 (53.3%) had received training on medication adherence, and 93 (61.2%) had seen the national guidelines for malaria treatment.

**Table 4:** Characteristics of the healthcare workers (n=152)

Characteristic	n (%)
Sex	
Female	108 (71.1)
Male	43 (28.3)
Missing	1 (0.7)
Age group, years	
20-29	61 (40.1)
30-39	59 (38.8)
40-49	18 (11.8)
≥50	14 (9.2)
Language usually spoken at home (multiple answers allowed)	
Lao	146 (96.1)
Language other than Lao	20 (13.2)
Missing	3 (2.0)
Place of work	
Health center	78 (51.3)
District hospital	61 (40.1)
Health office	13 (8.6)
Profession	
Nurse	51 (33.6)
Medical doctor	41 (27.0)
Community midwife	22 (14.5)
Primary health care	18 (11.8)
Medical assistant	9 (5.9)
Pharmacist or pharmacist assistant	6 (3.9)
Laboratory technician	5 (3.3)
Duration of working years; median (interquartile range)	7 (3-12)
Ever received training on malaria treatment	101 (66.4)
Ever received training on medication adherence	81 (53.3)
Ever seen the national guideline for malaria treatment	
Yes	93 (61.2)
No	39 (25.7)
Missing	20 (13.2)

The most common medication instruction that the participants gave malaria patients was the number of doses per day (85.5%) (Table 5), followed by the number of days of the regimen (74.3%), the side effects of the medicines (74.3%), and the importance of continuing to take the medicines (72.4%). Although the majority (67.1%) “always” or “often” confirmed with a patient whether he or she understood their instructions, 27.0% “occasionally” confirmed and 5.3% “never” confirmed the patient’s understanding. Regarding the experience of seeing or hearing about a non-adherent malaria patient, 125 participants (82.2%) had seen a malaria outpatient with poor adherence and 64 (42.1%) had heard about a malaria patient not completing the 3-day regimen of AL. The major reasons for patient non-adherence as perceived by the participants were patient illiteracy (42.2%), long distance to the healthcare facility (37.5%), and poor understanding of the instructions (31.3%). The majority (88.8%) understood that poor patient adherence to AL can cause drug-resistant malaria. According to the participants’ perception, the person responsible for managing patient adherence to medication was the healthcare worker who prescribes the medicines (69.7%), followed by the healthcare worker who hands the medicines over to the patients (62.5%) and the caregivers (45.4%).

**Table 5:** Practices, experiences and perceptions of the healthcare workers (n=152)

	n (%)
Contents of medication instructions	
Number of doses per day	130 (85.5)
Number of days of the regimen	113 (74.3)
Side effects of the medicine	113 (74.3)
Importance of continuing to take medicines	110 (72.4)
Effect of medicine	64 (42.1)
Number of tablets per dose	60 (39.5)
Frequency of confirming whether the patient understood how to take the medicines	
Never	8 (5.3)
Occasionally	41 (27.0)
Often	51 (33.6)
Always	51 (33.6)
Missing	1 (0.7)
Frequency of seeing malaria outpatients with poor adherence to medication instructions	
Never	26 (17.1)
Occasionally	113 (74.3)
Often	9 (5.9)
Always	3 (2.0)
Missing	1 (0.7)
Having heard of patients who did not correctly complete the 3-day regimen of AL	64 (42.1)
Perceived reasons that patients did not complete the regimen (multiple answers allowed) n=64	
Illiterate	27 (42.2)
Long distance between patient’s home and healthcare facility	24 (37.5)
Poor understanding of instructions	20 (31.3)
Low income	12 (18.8)
Mild symptoms	3 (4.7)
Not having health insurance	3 (4.7)
Fear of side effects	3 (4.7)
Others	7 (10.9)
Perceived effect of poor AL adherence on the emergence of drug-resistant malaria	
Poor adherence can cause drug-resistant malaria	135 (88.8)
Poor adherence cannot cause drug-resistant malaria	16 (10.5)
Missing	1 (0.7)
Perceived responsible person to manage medication adherence (multiple answers allowed)	
Healthcare workers who prescribed medicine	106 (69.7)
Healthcare workers who hand over medicines to patients	95 (62.5)
Caregivers	69 (45.4)
Patients	29 (19.1)
Others	6 (3.9)

The younger the healthcare workers were, the more they confirmed the patients’ understanding (*p* = 0.004) (Additional file 1). However, there was no statistically significant association between the duration of working and confirming patients’ understanding. Those who explained the importance of continuing to take medicines more often significantly confirmed the patients’ understanding than those who did not provide this explanation (*p* = 0.03).

## Discussion

In the present study of patients aged 18 years or older, the medication adherence rate was 94.4%, similar to the rates reported from previous studies in an Asian setting. A prospective observational study using AL (Coartem®) in Myanmar with patients of whom 25% were older than 14 years old reported an adherence rate of 89.5% [25]. A randomized controlled trial using AL (Coartem®) in Bangladesh showed an adherence rate of 93.1% in the non-directly observed treatment group, in which 54% of the patients were older than 14 years old [28]. Compared to these adherence rates for AL, that in the present study was slightly higher: The difference may be partly due to the difference in the characteristics of the study participants. Especially, the age difference could be critical as several studies showed that the AL adherence rate is likely to be higher in adults than in children [24, 28]. One of these studies was a prospective observational study and the other was a randomized controlled trial, but the non-directly observed treatment group was similar to a prospective observational study. Thus, there were no big differences between these studies in terms of the study design. Furthermore, these previous studies did not mention whether the patients were blinded to potential follow-up. Other attributable factors could be the study settings and differences in study implementation.

The non-adherent patients in the present study did not take all of the tablets because they felt better before finishing the regimen. This has been widely reported in a number of adherence studies as a major reason for not taking all of the AL tablets: two studies in Kenya and Uganda, both of which targeted young children [12, 13], and three studies in Uganda, Bangladesh, and Ethiopia [27–29], all of which targeted all age groups and used AL. The reason why the patients quickly feel better is related to the pharmacological characteristics of the medicine. Approximately 2 h after taking AL, the concentration of artemether reaches its peak, which reduces the asexual parasite mass and rapidly improves symptoms [30]. Lumefantrine is absorbed and cleared more slowly, which prevents recrudescence. Therefore, the reason for non-adherence found in the present study, i.e., improvement in physical condition, can be a major reason for not completing the AL regimen in the Lao setting.

The results of the patient study showed that all of the non-adherent patients were explained the importance of completing the prescribed AL, and they perceived that the instructions were clear. Therefore, there may be at least two possible explanations for non-adherence in the present study: One is that the healthcare workers in charge of the patients failed to emphasize the need for completing the medication regimen regardless of the improved physical condition. The other is that the non-adherent patients did not understand the medication instructions well due to linguistic reasons. Almost all of the malaria patients were Mangkong, whereas the healthcare workers were Lao. The difference in mother tongues between them may have prevented the patients from understanding the medication instructions clearly. In a Zambian study that assessed adherence to the combination of sulphadoxine-pyrimethamine and artesunate, giving medication instructions to caregivers in their mother tongue lowered the risk of non-adherence [31]. A qualitative study in Lao PDR that analyzed the constraints in implementing the strategies for maternal, neonatal, and child health services revealed that a lack of language skills among healthcare workers was a common constraint [32].

The results of the healthcare workers study showed that 74.3% of the healthcare workers occasionally saw malaria outpatients who poorly adhered to medication instructions. Additionally, 42.1% of the healthcare workers had heard of a patient who did not complete the 3-day regimen of AL. These results suggested that patients who do not adhere to the AL regimen are sometimes seen in the study districts. According to the healthcare workers’ perception, the major reasons for non-adherence were “illiteracy,” “poor understanding of the instructions,” and “long distance from the patient’s home to healthcare facility.” These results suggest that it is important to improve communication between patients and the dispensing healthcare workers. All but one of the healthcare workers in the present study depended solely on oral medication instructions. To facilitate a better understanding of medication instructions by malaria patients in a rural Lao setting where many malaria patients are illiterate, we recommend that healthcare workers supplement oral instructions with visual instructions. In a randomized controlled trial using a 3-day regimen of chloroquine in Nigerian children with *P. falciparum* malaria, the use of visual media (i.e., a pictorial insert) improved clinical outcomes [33]. In a qualitative study conducted in remote, linguistically isolated villages in Nong district, where the present study was also conducted, posters were used effectively for villagers to understand the concept of symptomatic and asymptomatic malaria [18]. This experience could be applied to medication instructions. In the case of AL, packaging of the brand-name drug (Coartem®) has been designed to educate the patient. Each dose is surrounded by a frame, and when and how many tablets the patient should take is designated with a picture. Especially, the Coartem® Dispersible (for children) pack has pictures showing that each dose has the effect of killing parasites, which might be helpful for patients in understanding the meaning of completing the regimen. In contrast, the package for the generic medicine used during the study period is simpler; each dose is packaged separately without a surrounding frame printed on the front side, and it has six frames printed only on the back side. Thus, packaging design should be considered when selecting the kind of AL to be dispensed.

The results of the healthcare workers study also showed that 27.6% of the workers did not regularly tell a patient the importance of taking all prescribed AL tablets, and 32.2% did not often or always confirm the patients’ understanding of how to take the medicine. When comparing the healthcare worker who confirmed patients’ understanding with those who did not, the younger healthcare workers were more likely to confirm the patients’ understanding. In a previous study that examined the factors affecting adherence to national malaria treatment guidelines among healthcare workers in Uganda, the age of the healthcare workers and their duration of employment were not found to be associated with adherence [34]. In another study in Malawi, the odds of appropriate malaria treatment were increased as healthcare worker age increased [35]. This result of the present study was not consistent with that of these other studies. However, there was no statistically significant association between adherence and the duration of working in the present study, so further investigation is needed. Those healthcare workers who explained the importance of continuing to take medicines more often tended to confirm the patients’ understanding than those who did not. It is surmised that those workers who gave clear explanations likely also addressed the various aspects of medication adherence.

Such lack of instruction could have contributed to the poor medication adherence seen in the study districts. Thus, the Lao National Malaria Control and Elimination Program should ensure that every healthcare worker who is involved in the treatment of malaria provide all essential instructions to their patients. Previous studies have suggested that intensifying healthcare workers compliance to malaria treatment guidelines is needed to properly implement the guidelines to improve malaria treatment [36, 37].

The present study has some limitations. First, the surveyors were allowed to skip the observation of a leftover tablet in the AL packaging sheet if they were unable to visit the patient’s home on the follow-up day. Therefore, for the eight patients who were surveyed over the phone, the assessment of the adherence rate depended solely on the honesty of the patients’ self-reporting. Second, some healthcare workers in the study health centers also worked as surveyors for the present study because of limited human resources at the study site. The quality of care including dispensing practices of healthcare workers might have been improved as seen in the previous study [38] because of recognition of appropriate dispensing and counseling through informed consent and the questionnaire during the patient study. Third, the patients were explained that they would be followed up within several days after written informed consent to participate in the study was obtained from them. Thus, some patients might have improved their adherence to the regimen to show better behavior. Fourth, the number of the patients was limited, and most of them were treated at two study facilities. Therefore, the findings of the present study could not exactly reflect the situation of the study districts. Fifth, some malaria patients were followed up on day 2, and thus, there is a possibility that they became non-adherent after the follow-up survey. Sixth, the difference in the medication package is likely to have some impact on patients’ adherence [39, 40]. However, we cannot estimate the magnitude of the impact because we did not confirm which package was prescribed to which patient. Finally, in the study districts, the patient study was aimed at selected healthcare facilities, whereas the healthcare workers study was aimed at all of the healthcare facilities. Therefore, the present research could not exactly link the findings obtained from these two studies. Considering these limitations, the adherence rate observed in the present study could be overestimated [41, 42].

## Conclusions

At 94.4%, the rate of patient adherence was high in the study districts. Among the non-adherent patients, the reason for their non-adherence was improvement in their physical condition. Most of the participants (74.3%) in the healthcare workers study who gave medication instructions to malaria patients reported that they occasionally saw a malaria patient who poorly adhered to the instructions. Therefore, based on the healthcare workers’ experience and the study methodology, it is likely that the observed adherence rate could be overestimated. The participants in the healthcare workers study perceived that the major reasons for poor adherence were attributable to the patients, i.e., their poor understanding of the instructions and illiteracy. In fact, there were linguistic differences between the patients and healthcare workers in the patient study. However, poor adherence also appeared to be attributable to the healthcare workers: 27.6% of them did not regularly instruct the patient on the importance of completing the prescribed AL tablets, and 32.2% did not often or always confirm the patients’ understanding of the medication instructions. Thus, the Lao National Malaria Control and Elimination Program should ensure that dispensing healthcare workers tell patients to complete the AL regimen regardless of the patient’s improvement in physical condition, and they should also confirm patient understanding of the medication instructions.

## Additional file

Additional file 1: **Table S1.** Comparison between the healthcare workers who often/always confirmed patients’ understanding and those who never/occasionally confirmed (*n* = 151) (DOCX 46 kb)
