# Supplementary material for: Patients’ adherence to artemisinin-based combination therapy and healthcare workers’ perception and practice in Savannakhet province, Lao PDR
Source: Trop Med Health. 2018 Dec 22;46:44. doi: 10.1186/s41182-018-0125-6 (PMC6303952; doi:10.1186/s41182-018-0125-6)
Supplement: Supplementary file 1 — Table S1. Comparison between the healthcare workers who often/always confirmed patients’ understanding and those who never/occasionally confirmed (n = 151) (DOCX 46 kb) [file 41182_2018_125_MOESM1_ESM.docx]

**Additional file 1**

**Table S1 Comparison between the healthcare workers who often/always confirmed patients’ understanding and those who never/occasionally confirmed (n=151)**

| Characteristic | | | | Often/  Always  (n=102)  n (%) | | Never/  Occasionally  (n=49)  n (%) | | p value | |
| --- | --- | --- | --- | --- | --- | --- | --- | --- | --- |
| Sex | | | |  | |  | |  | |
|  | Female | | | 75 (70.1) | | 32 (29.9) | | 0.386 | |
|  | Male | | | 27 (62.8) | | 16 (37.2) | |  | |
| Age group, years | | | |  | |  | |  | |
|  | 20-29 | | | 48 (76.2) | | 13 (20.6) | | 0.004 | |
|  | 30-39 | | | 39 (66.1) | | 20 (33.9) | |  | |
|  | 40-49 | | | 8 (47.1) | | 9 (52.9) | |  | |
|  | ≥50 | | | 7 (50.0) | | 7 (50.0) | |  | |
| Place of work | | | |  | |  | |  | |
|  | District hospital | | | 38 (63.3) | | 22 (36.7) | | 0.511 | |
|  | Health center | | | 56 (71.8) | | 22 (28.2) | |  | |
|  | Health Office | | | 8 (61.5) | | 5 (38.5) | |  | |
| Profession | | | |  | |  | |  | |
|  | Nurse, Medical doctor, Medical assistant Pharmacist, pharmacist assistant | | | 75 (70.8) | | 31 (29.2) | | 0.197 | |
|  | Community midwife, Primary health care, Laboratory technician | | | 27 (60.0) | | 18 (40.0) | |  | |
| Duration of working years; median (interquartile range) | | | | | |  | |  | |
| 6 (3-11.75) | | | | | | 7.5 (4-24.5) | | 0.12 | |
| Ever received training on malaria treatment | | | |  | |  | |  | |
|  | Yes | | | 66 (66.0) | | 34 (34.0) | | 0.357 | |
|  | No | | | 36 (73.5) | | 13 (26.5) | |  | |
| Ever received training on medication adherence  Yes | | | | 54 (67.5) | | 26 (32.5) | | 0.386 | |
|  | No | | | 47 (67.1) | | 23 (32.9) | |  | |
| Ever seen the national guideline for malaria　treatment | | | | | |  | |  | |
|  | Yes | | | 65 (69.9) | | 28 (30.1) | | 0.715 | |
|  | No | | | 26 (66.7) | | 13 (33.3) | |  | |
| **Contents of medication instructions** | | | | |  | |  | |  |
| Number of pills per dose | | | Yes | | 38 (63.3) | | 22 (36.7) | | 0.369 |
|  | | | No | | 64 (70.3) | | 27 (29.7) | |  |
| Number of doses per day | | | Yes | | 88 (68.2) | | 41 (31.8) | | 0.671 |
| No | | 14 (63.6) | | 8 (36.4) | |  |  |  |  |
| Number of days of treatment | | | Yes | | 79 (69.9) | | 34 (30.1) | | 0.285 |
| No | | 23 (60.5) | | 15 (39.5) | |  |  |  |  |
| Effect of the medicines | | | Yes | | 43 (67.2) | | 21 (32.8) | | 0.935 |
| No | | 59 (67.8) | | 28 (32.2) | |  |  |  |  |
| Side Effect of the medicines | | | Yes | | 81 (71.7) | | 32 (28.3) | | 0.061 |
| No | | 21 (55.3) | | 17 (44.7) | |  |  |  |  |
| Importance to continue taking medicines | | | | |  | |  | |  |
|  | | | Yes | | 80 (72.7) | | 30 (27.3) | | 0.03 |
|  | | | No | | 21 (53.8) | | 18 (46.2) | |  |
| Frequency of seeing malaria outpatients with poor adherence to medication instructions | | | | | | | | | |
|  | | | Never | | 17 (65.4) | | 9 (34.6) | | 0.259 |
|  | | | Occasionally | | 74 (65.5) | | 39 (34.5) | |  |
|  | | | Often | | 9 (100) | | 0 (0) | |  |
|  | | | Always | | 2 (66.7) | | 1 (33.3) | |  |
| Having heard of patients who did not correctly complete the 3-day regimen of AL | | | | | | | | | |
|  | | | Yes | | 42 (66.7) | | 21 （33.3） | | 0.845 |
|  | | | No | | 6 (68.2） | | 28 （31.8） | |  |
| **Perceived effect of poor ACT adherence on the emergence of drug-resistant malaria** | | | | | | | | | |
| Poor adherence can cause drug-resistant malaria | | | | | 90 (67.2) | | 44 (32.8) | | 0.898 |
| Poor adherence cannot cause drug-resistant malaria | | | | | 11 (68.8) | | 5 (31.3) | |  |
| **Perceived responsible person to manage medication adherence** | | | | | | | | |  |
| Healthcare workers who prescribed medicine | | | | |  | |  | |  |
|  | | | Yes | | 68 (64.2) | | 38 (35.8) | | 0.260 |
|  | | | No | | 31 (72.8) | | 11 (26.2) | |  |
| Healthcare workers who hand over medicines to patients | | | | | | |  | |  |
|  | | Yes | | | 65 (68.4) | | 30 (31.6) | | 0.652 |
|  | | No | | | 35 (64.8) | | 19 (35.2) | |  |
| Caregivers | | Yes | | | 51 (73.9) | | 18 (26.1) | | 0.101 |
|  | | No | | | 49 (61.3) | | 31 (38.8) | |  |
| Patients | | Yes | | | 16 (57.1) | | 12 (42.9) | | 0.202 |
|  | | No | | | 85 (69.7) | | 37 (30.3) | |  |
